# Respiratory Disease following Illicit Injection of Silicone: A Case Report

**DOI:** 10.1155/2013/743842

**Published:** 2013-07-15

**Authors:** Alex Charles Essenmacher, Seyed Amin Astani

**Affiliations:** ^1^Wayne State University School of Medicine, 540 East Canfield Street, Detroit, MI 48201, USA; ^2^Radiology Department, Henry Ford Health System, Wayne State University School of Medicine, 2799 West Grand Boulevard, Detroit, MI 48202, USA

## Abstract

Unregulated, pseudomedical procedures risk serious sequelae even when otherwise safe compounds are used. Silicone is commonly used legally in cosmetic procedures owing to its durability, resistance to heat and aging, and low immunogenicity. However, inappropriate or illegal silicone injection can pose severe local and systemic complications including serious pulmonary compromise. We describe the case of a 30-year-old female who presented with hemoptysis and progressive shortness of breath following illicit silicone injections to the gluteal fat and was found to have new, diffuse, bilateral, ground-glass opacities on contrast-enhanced pulmonary computed tomography. Transbronchial biopsy elucidated that this was a lipoid pneumonia-type injury secondary to silicone infiltration.

## 1. Introduction

Liquid silicone is a synthetic polymer incorporating oxygen and the semimetallic element silicon. It is widely used in plastic and reconstructive surgery as it displays little change in physical characteristics with temperature and age, is poorly immunogenic, and is not carcinogenic [1]. For most of its legitimate medical uses, silicone should be sequestered from soft tissue. If the silicon-containing material is exposed to soft tissue, it will generate a fibroblastic response and gradually increase the local volume. Silicone injections are therefore tempting to some patients seeking enhancement of the buttocks or breasts even when offered by unlicensed practitioners.

## 2. Case Presentation

A 30-year-old female smoker presented to the hospital with the chief complaint of hemoptysis two days after liquid silicone injections to the buttocks for cosmetic augmentation. She reported progressive shortness of breath one hour after the procedure.

On presentation, she was in significant respiratory distress and required 2 L via nasal cannula to maintain oxygen saturation above 90%. Vital signs obtained in triage included a blood pressure of 99/56 mmHg and respiratory rate of 20 per minute. She presented with tachycardia of 114 beats per minute, but during admission her rate stabilized between 70 and 100 bpm. She was afebrile throughout hospitalization. Formal pulmonary function testing demonstrated a restrictive disease pattern with a forced vital capacity (FVC) of 2.19 L (63% of predicted), forced expiratory volume in 1 second (FEV1) of 1.59 L (54% of predicted), and an FEV1/FVC ratio of 72.6. 

**Figure 1:**
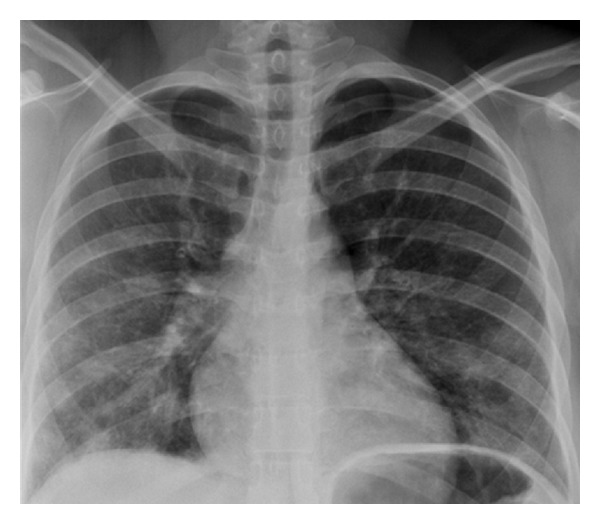
A 30-year-old female after illicit silicone injection. Findings: PA chest radiograph demonstrates diffuse haziness throughout the middle and lower lungs especially in the right lower lung.

**Figure 2:**
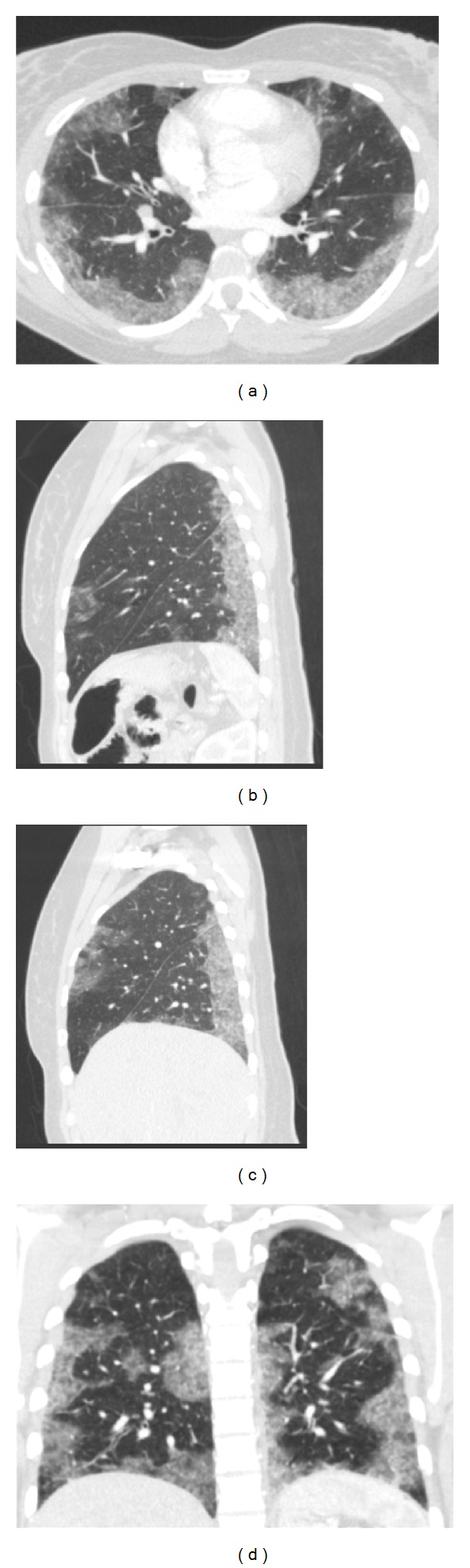
A 30-year-old female after illicit silicone injection. Findings: CT pulmonary angiography (axial (a), left sagittal (b), right sagittal (c) and coronal (d)) demonstrates diffuse, bilateral, ground-glass opacities greater on the right than on the left and predominantly at the lung bases.

**Figure 3:**
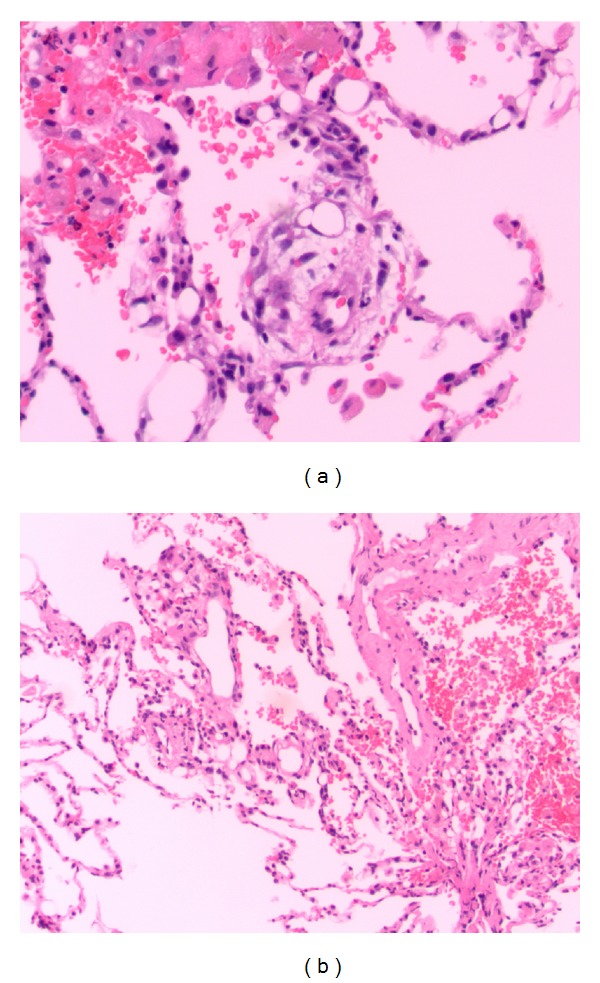
Pathologic specimens obtained during bronchoscopy from a 30-year-old female after illicit silicone injection to subcutaneous tissue. Findings: Silicone infiltration with subsequent spillage into alveolar spaces; exogenous lipoid pneumonia-type injury pattern.

There was a D-dimer elevation to 680 ng/mL (reference: <490 ng/mL). An initial chest radiograph demonstrated diffuse haziness throughout the middle and lower lungs more pronounced in the right lower lobe (Figure 1). Pulmonary CT angiography was negative for an acute embolus but demonstrated bilateral, peripheral ground-glass opacities more prominent toward the bases (Figure 2). Bronchoalveolar lavage twelve hours later demonstrated sanguineous fluid with no change throughout suctioning. Transbronchial biopsies revealed an injury pattern consistent with exogenous lipoid pneumonia and silicon vacuoles (Figure 3).

 Methylprednisolone sodium succinate was initiated at a dose of 125 mg every six hours. Within 48 hours full resolution of her shortness of breath was attained clinically, and her oxygen saturation remained above 94% without supplemental oxygen. The patient was discharged on hospital day 5 in good condition. Her discharge prescriptions included prednisone 40 mg oral once daily and an albuterol inhaler as needed for dyspnea.

At her follow-up appointment with the pulmonologist two weeks later, significant improvement was noted on a repeat pulmonary function test: FVC: 3.36 L (97% of predicted); FEV1: 2.57 L (88% of predicted); and FEV1/FVC: 76.5. The patient had no persistent respiratory complaints, and the lungs were clear to auscultation bilaterally with no crackles, rhonchi, or wheezes. No follow-up images were obtained.

## 3. Discussion

Liquid silicone is a synthetic polymer incorporating oxygen and the semimetallic element silicon. It has been judiciously used by licensed practitioners in the United States for more than fifty years. However, a demand also exists for cheaper augmentation procedures performed by unlicensed and possibly unskilled practitioners. A potential for adverse events, including respiratory compromise, infection, and death, exists, and currently the United States Food and Drug Administration approves injected liquid silicone only in certain indications intraocularly.

The local effect of injected liquid silicone can include tissue necrosis, foreign body giant cell reactions, and infection [2]; severe local reactions are common [3]. Silicone has also been demonstrated in distant organs following subcutaneous administration suggesting an embolic phenomenon manifesting as regional lymphadenopathy, granulomatous hepatitis [4], interstitial nephritis, and other acute, systemic illnesses [5], but pulmonary and neurologic sequelae especially warrant emergency attention.

The respiratory consequences have been described as severe pulmonary toxicity, acute pneumonitis [1, 6–8], acute respiratory distress syndrome, and pulmonary embolism [9]. Pulmonary manifestations of subcutaneous silicone injections are proposed to result from inadvertent injection of silicone directly into a vein, increased tissue pressures at injection sites, and from local massage or trauma at the site. The phenomenon is acute with symptoms presenting within 72 hours after injection in most cases, and the risk appears to increase with higher doses of silicone. The pathologic findings on lung biopsy include alveolar hemorrhage [10–12], silicone deposits within the alveoli [13], and inclusions within alveolar macrophages [7, 14, 15]. The biopsy of our patient revealed silicone infiltration with subsequent spillage into alveolar spaces. The response induced is similar to the injury pattern seen in exogenous lipoid pneumonia, a pathology traditionally resulting from inhalation of oily (liquid fat) substances.

When presenting with primarily respiratory complaints such as dyspnea and hemoptysis, the silicone-induced pathology broadly overlaps with the fat embolism syndrome clinically and radiologically [11]. Chest radiographs often demonstrate bilateral, patchy infiltrates. The computed tomography (CT) findings of an acute pneumonitis pattern of silicone injury include peripheral, ground-glass opacities and low attenuation in affected lung; these findings resemble acute respiratory distress syndrome and bronchiolitis obliterans organizing pneumonia [16].

Survival is likely, and treatment involves ventilation if necessary and steroids with uncertain utility. Zamora et al. [8] reported five cases of silicone-associated pneumonitis that all recovered with steroid treatment but admit that steroid responsiveness cannot be proven as there were no untreated patients with whom outcomes could be compared. Steroid therapy is often initiated in the setting of unexplained acute respiratory failure. A less common manifestation of acute embolic events due to silicone involves neurologic symptoms, including altered consciousness and coma, and is rapidly fatal [11].

## 4. Conclusion

Silicone pneumonitis and the fat embolism syndrome are similar clinically and radiographically. Dyspnea, hypoxemia, and hemoptysis are possible manifestations, while chest radiographs often demonstrate bilateral, patchy infiltrates. Peripheral, ground-glass opacities and low CT attenuation in affected lung also resemble acute respiratory distress syndrome and bronchiolitis obliterans organizing pneumonia. The diagnosis can frequently be missed with a wide differential diagnosis by radiology combined with often incomplete patient history. Bronchoalveolar lavage and tissue biopsy can help make the diagnosis.
